# The Frequency and Clinical Significance of *IDH1* Mutations in Chinese Acute Myeloid Leukemia Patients

**DOI:** 10.1371/journal.pone.0083334

**Published:** 2013-12-20

**Authors:** Lixun Guan, Li Gao, Lili Wang, Meng Li, Yue Yin, Li Yu, Chunji Gao

**Affiliations:** 1 Department of Hematology and Hainan Branch, General Hospital of PLA, Beijing, China; 2 Department of Hematology, China-Japan Friendship Hospital, Beijing, China; University of Texas M.D. Anderson Cancer Center, United States of America

## Abstract

**Objective:**

Mutations in the gene encoding isocitrate dehydrogenease 1 (*IDH1*) occur in various hematopoietic tumors including acute myeloid leukemia (AML), myeloproliferative neoplasms and myelodysplastic syndromes. *IDH1* mutations are significant in both diagnosis and prognosis of these conditions. In the present study we determined the prevalence and clinical significance of *IDH1* mutations in 349 samples from newly diagnosed AML patients.

**Results:**

Of the 349 AML patient specimens analyzed, 35 (10.03%) were found to have *IDH1* mutations including 4 IDH1 R132 mutations and 31 non-R132 mutations. IDH1 non-R132 mutations were largely concentrated within AML-M_1_ (35.72%, *p*<0.01). We identified five *IDH1* mutations that were novel to AML: (1) c.299 G>A, p.R100Q; (2) c.311G>T, p.G104V; (3) c.322T>C, p.F108L; (4) c.356G>A, p.R119Q; and (5) c.388A>G, p.I130V. In addition, we identified three *IDH1* mutations that were previously described in AML. The frequency of *IDH1* mutations in AML patients with normal karyotype was 9.9%. *IDH1 non-R132* mutations were concurrent with mutations in *FLT3-ITD* (*p*<0.01), *CEBPA* (*p*<0.01), and *NRAS* (*p*<0.01), as well as the overexpression of *MN1* (*p*<0.01) and *WT1*(*p*<0.01). The overall survival (OS) in the patients with *IDH1 non-R132* mutations compared to patients without *IDH1* mutations don't reach statistically significance (median 521 days vs median: not reached; n.s.).

**Conclusion:**

*IDH1 non-R132* mutations occurred frequently in newly diagnosed adult Chinese AML patients, and these mutations were associated with genetic alterations. The OS was not influenced by *IDH1 non-R132* mutations in the present study.

## Introduction

Acute myeloid leukemia (AML) is a hematopoietic malignancy caused by mutations in clonal multipotent stem cells or early myeloid progenitor cells. This disease is extremely diverse with regards to clinical manifestation, prognosis and outcome. The establishment and development of the MICM (Morphology, Immunology, Cytogenetics, Molecular biology) diagnostic platform, has shown that cytogenetics and molecular biology were significant for diagnosis and prognosis. Although some AML patients lack distinctive chromosomal abnormalities, molecular biological studies have demonstrated that certain genetic mutations and abnormalities in gene expression are related to AML prognosis. In 2008, the WHO classification of AML was revised to include *NPM1* and *CEBPA* [1], and in the clinical settings their mutational status impact risk classification, prognostic judgment and therapeutic choice.

The *IDH1* gene is located on chromosome 2 at 2q33.3. In the TCA cycle (tricarboxylic acid cycle), *IDH1* catalyzes oxydehydrogenation of isocitrate to yield the intermediate oxalosuccinic acid that is further transformed into α-ketoglutarate (α-KG) via oxidative decarboxylation, while reducing nicotinamide adenine dinucleotide (NAD^+^) or nicotinamide adenine dinucleotide phosphate (NADP^+^) to yield NADH or NADPH. *IDH1* proteins are mainly localized to the cytoplasm and peroxisome. Mutations in *IDH1* impairs the affinity of the enzyme for its substrate, thereby inhibiting its enzymatic activity leading to decreased formation of α-KG and increased HG production.

*IDH1* mutations were identified in AML patients with normal karyotype AML using whole genome sequencing[2]. In this study, *IDH1* mutations alone had no significance on the independent prognosis for AML outcome, but better prognosis was achieved when *IDH1* and *NPM1* mutations occurred simultaneously. This study sparked research into the field of *IDH1* mutations within various AML populations, particularly the frequency of mutations and their clinical significance. To date there the approximate rate of *IDH1* mutations in Western countries including the United States[3], [4], [5], Canada[6], France[7], Germany[8], [9], [10], [11], Netherlands[12] and England[13], [14] is 10–14%. However, there are limited reports regarding the rate of *IDH1* mutations in Chinese AML patients suggest a mutation rate of 2–6.3%. The main goal of the present study was to evaluate the *IDH1*-related incidence of AML in newly diagnosed adult Chinese AML patients, as well as explore the correlation between *IDH1* mutations and the clinical index, cytogenetics, molecular biology or prognosis.

### Ethics Statement

This study has been approved by the ethics committee of Chinese PLA General Hospital. Written informed consent was obtained from Adult parents or guardians of children who were enrolled in this study.

## Patients and Methods

### Patients

All of the patients used in this study provided a written informed consent of enrollment. The adult AML patients (n = 349) were admitted to outpatient and inpatient services of Department of Hematology, Chinese PLA General Hospital between January 2005 and October 2011. Among them, 214 patients were male and 135 female, and median age was 41 y (range of 10–88 y). The specimens were obtained prior to the patients receiving any treatments for AML and after the patients reaching remission. The specimens were also got from 10 healthy volunteer as control.

### Morphological examination of myeloid cells

#### Myeloid cell smear

After regular Wright-Giemsa staining, 250 karyotes were counted under a light microscope and the cellular morphology and ratio in different stages were analyzed. The megakaryocytes on the whole slide were enumerated and the morphology of at least 25 megakaryocytes was analyzed. In the peripheral blood smear, the leucocytes were classified and morphology of platelets was analyzed.

#### Cytogenetic examination

Heparin-treated bone marrow (5 ml) was extracted and the myeloid cells were counted. The chromosomal specimens were prepared at a cell density of 1 to 2×10^6^/ml using the direct method, and then were processed by the R banding method. The karyotype was described in accordance with “An International System for Human Cytogenetic Nomenclature” (ISCN 2005).

### Detection of genetic mutations and analysis of gene expression

Mutational analysis of *NRAS, NPM1, CEBPA, IDH1, FLT3-ITD, RUNX1* and *MLL* was performed via DNA sequencing. Table S1 lists the primers used for DNA sequencing. Quantitative analysis of gene expression for *WT1, PRAME, EVI1, KIT, MN1* and *FLT3* were conducted using the TaqMan-MGB-probe approach. The screening of the AML-related genes was carried out by nested PCR with the primers and probes listed in Table S2. The definition of Gene Overexpression is that target gene/abl ≥80%. The design of primers of IDH1 is based on cDNA.

### Statistical analysis

Overall survival (OS) was defined as the period between confirmed diagnosis and death of the patient or between confirmed diagnosis and the date of last follow-up for patients that were still alive. Kaplan-Meier analysis was conducted to compare the OS differences between the patient groups. The median follow-up time was obtained by calculating last follow-up date of the surviving patients and the date of death for the dead patient. The correlation between *IDH1* mutations and cytogenetic or molecular biological parameters were determined by a Chi square (X2) test. The difference between the *IDH1* mutation group and other groups was measured using the Fisher exact probability method. The continuous variables were tested using a Mann-Whitney U test. The normal continuous variables were described with mean and standard deviation, while the non-normal continuous data were described with median and range. The *t* test was used for comparison between the groups of the normal continuous variables, and the rank-sum test was used for comparison among groups for non-normal continuous variables.

### Treatment plan

Induction therapy for non-M3 AML consistes of standard DA or HA regime, and M3 is treated with ATRA with/without AsZO3. Patients not entering CR take other induction therapies. All CR patients received an early consolidation therapy with AraC. Allo-sct or auto-sct treatment was risk-adapted.

## Results

### 1. Clinical features of AML patients with *IDH1* mutations

Of the 349 newly diagnosed adult Chinese AML patients, 35 patients (10.03%) had *IDH1* mutations, including 4 *IDH1* R132 mutation patients and 31 IDH1 non-R132 mutation patients. There was no statistically significant difference in the occurence of *IDH1* R132 mutations and non-R132 mutation between male and female patients. The median age of the patients with two *IDH1* mutations groups were not statistically significantly different than the median age in *IDH1* wild type group. In addition, there were no statistically significant differences between the two *IDH1* mutation groups and wild type group with regards to leucocyte count (*n.s.*), hemoglobin (*n.s.*), platelet count (*n.s.*) and the ratio of blast cells (*n.s.*). The demographic and clinical features for the patients are detailed in Table 1.

**Table 1:** Patient demographics, clinical data and cytogenetic changes in AML patients with/without *IDH1* mutations.

Parameters	Total	R132 IDH1-mutated	Non-R132 IDH1-mutated	IDH1 Wild type	P-value (R132 v wt)	P-value (non-R132 v wt) Wt
Patients (n)	349	4	31	314		
Female	135	2	7	126	1	0.055
Male	214	2	24	188		
Age	41 (10–88)	40(21–64)	41(14–66)	41 (10–88)	0.883	0.989
Leucocytes (×109)	35 (0.26–157)	14(2.99–20.1)	56(1.07–456)	35 (0.26–157)	0.562	0.121
Hemoglobin(g/L)	84 (2.81–167)	109(98–118)	90(38–131)	83 (2.81–167)	0.077	0.166
Platelets (×109)	71 (0–297)	333(18–297)	71(11–278)	68 (0–297)	0.216	0.641
Blast cells	0.6 (0.2–0.99)	0.65(0.38–0.9)	0.61(0.2–0.99)	0.6 (0.2–0.99)	0.679	0.752
M0	1/0.3%	0	0	1(100%)		
M1	14/4.0%	0	5(35.7%)	9(64.3%)	0.311	0.005
M2	114/32.7%	2(1.4%)	9(7.9%)	105(92.1%)	0.001	0.355
M3	44/12.6%	0	3(6.8%)	41(93.2%)	0.046	0.624
M4	74/21.2%	1(1.4%)	5(6.8%)	68(93.2%)	0.008	0.309
M5	76/21.8%	1(1.3%)	7(9.2%)	68(90.8%)	0.008	0.821
M6	24/2.9%	0	2(8.3%)	22(91.7%)	0.211	1
M7	0	0	0	0		
Normal karyotype	151/43.3%	3(2%)	12(7.9%)	136(90.1%)	0	0.305
Abnormal karyotype	192/55. 0%	1(0.5%)	19(9.9%)	172(89.6%)		
t(15;17)	28/8.2%	0	2(7.1%)	26(92.9%)	0.153	0.840
t(8;21)	29/8.5%	0	4(13.8%)	25(86.2%)	0.165	0.702
Inv16/t(16;16)	12/3.5%	0	0	12(100%)	0.491	0.491
+8	14/4.0%	1(7.1%)	2(14.3%)	11(78.6%)	1	0.853
t(7;11)	8/2.3%	0	1(12.5%)	7(87.5%)	0.797	1
del(7)	23/6.7%	0	2(8.7%)	21(91.3%)	0.229	1
del(5)	19/5.5%	0	1(5.3%)	18(94.7%)	0.293	0.75
Others	59/17.2%	0	7(11.9%)	52(88.1%)	0.018	0.607

### 2. Distribution features of *IDH1* mutations

**Figure 1:**
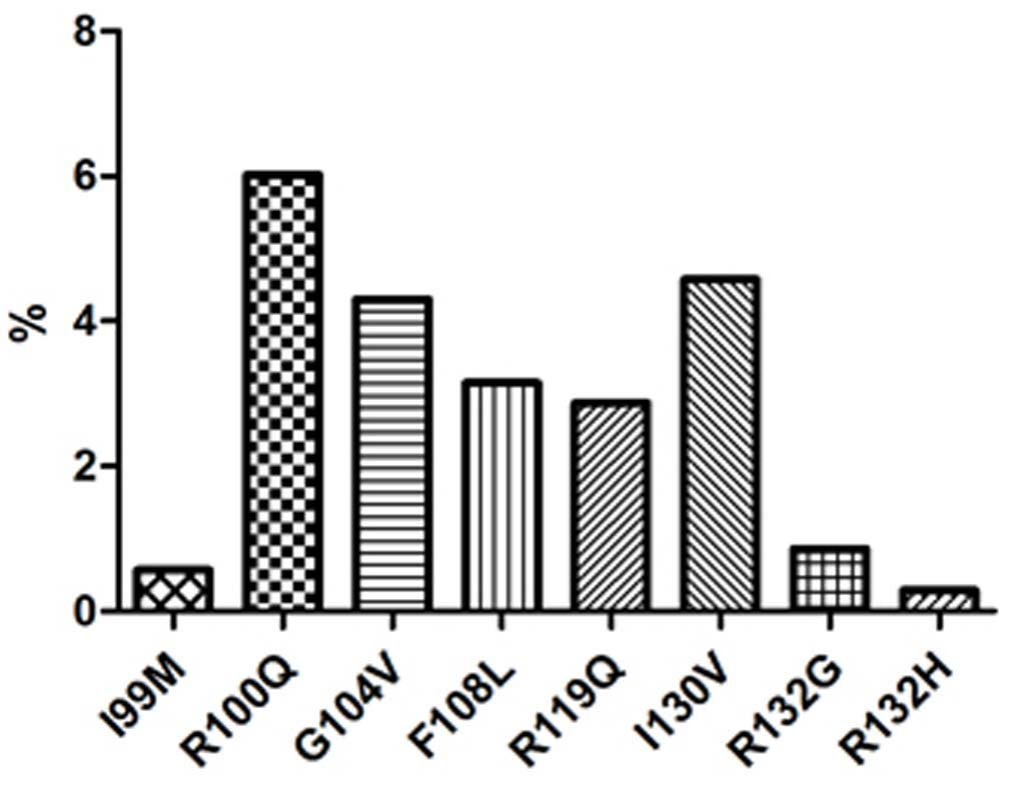
Incidence of different subtypes of *IDH1* mutations. Eight *IDH1* mutations were detected in 35/349 patients.

**Figure 2:**
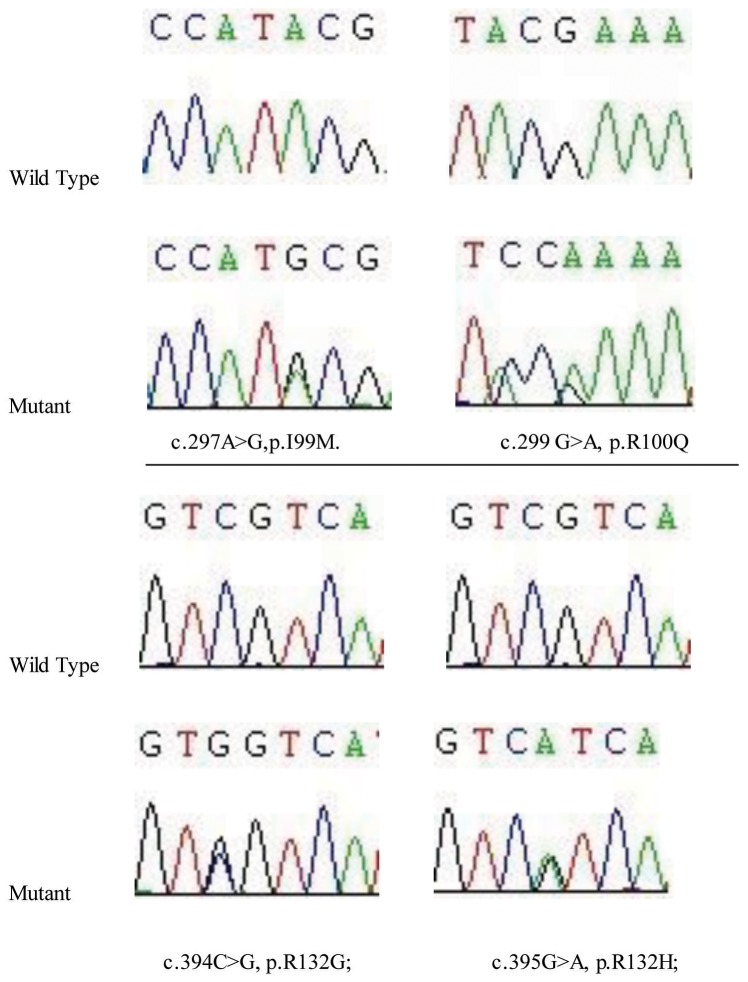
DNA sequencing of *IDH1* mutations. DNA sequencing chromatograms of representative heterozygous *IDH1* mutations and reference wild type *IDH1*.

We identified eight different nonsynonymous *IDH1* mutations: (1) c.297A>G, p.I99M; (2) c.299 G>A, p.R100Q; (3) c.311G>T, p.G104V; (4) c.322T>C, p.F108L; (5) c.356G>A, p.R119Q; (6) c.388A>G, p.I130V; (7) c.394C>G, p.R132G; and (8) c.395G>A, p.R132H. The percentage of each mutation was 0.57%, 6.02%, 4.3%, 3.15%, 2.87%, 4.58%, 0.86%, and 0.29%, respectively (Fig. 1). To the best of our knowledge, five of these *IDH1* mutations (c.299 G>A, p.R100Q; c.311G>T, p.G104V; c.322T>C, p.F108L; c.356G>A, p.R119Q; and c.388A>G, p.I130V) are reported here for the first time in AML, but not in general[15], [16], [17]. The Sanger traces for all novel IDH1 mutations were listed in Figure S1. After examining matched remission specimens and 80 healthy volunteer' specimens, no variant are found, so it is proved that these are novel mutations rather than polymorphism. All mutations were heterozygous (Fig. 2). The p.R100Q mutation occurred at the substrate binding site and was shared the identical role as its analogous residue of IDH2 p.R140 in tumorigenesis according to the structural modeling for IDH1.

### 3. The correlation between *IDH1* mutations and FAB types

Among IDH1 R132 mutation, the majority was classified as AML M2(2/114) and M4(1/74) followed by M5(1/76) based on the FAB classification system. While the frequency of *IDH1* non-R132 mutations were divided into 6 subtypes of AML: M_1_ 35.7% (5/14), M_2_ 7.9% (9/114), M_3_ 6.8% (3/44), M_4_ 6.8% (5/74), M_5_ 9.2% (7/76) and M_6_ 8.3% (2/24). There were no *IDH1 non-R132* mutations detected in M_0_ and M_7_, and *IDH1 non-R132* mutations occurred most frequently in M_1_ (5/14; 35.7%, *p*<0.01). However, there were no statistic differences compared with other subtypes (Table 1).

### 4. The correlation between *IDH1* mutations and karyotypes

Karyotype analyses were carried out in 343 of 349 patients (Table 1). Of the 343 cases, 151 (44%) cases showed normal karyotypes and 192 (56%) cases showed abnormal karyotypes. Among the 4 *IDH1* R132 mutations, only one showed abnormal karyotype (+8). On the other hand, 19 of the 31 *IDH1* non-R132 mutations showed abnormal karyotype, 2 cases with t(15;17), 4 cases with t(8;21),2 cases with +8,1 cases with t(7;11),2 cases with del(7), and 1 cases with del(5 q).

### 5. The correlation between *IDH1* mutations and other genetic abnormalities

Wild type *IDH1* was largely not associated with other commonly detected genetic abnormalities (Table 2). Due to the fewer case number of *IDH1* R132 mutations, the correlation between *IDH1* R132 and other genes was not took into account. *IDH1* non-R132 mutations were often accompanied with mutations in *FLT3-ITD* (*p*<0.01), *NRAS* mutation (*p*<0.05) *and CEBPA* (*p*<0.01), as well as overexpression of *WT1* (*p*<0.01) and *MN1* (*p*<0.01). Other genetic mutations were detected along with *IDH1* non-R132 mutations, but there was no statistically significant difference between the *IDH1* non-R132 mutant group and wild type group. Among the 35 patients with *IDH1* mutations, 3 cases occurred independently of other genetic mutations, 20 cases had an additional mutation in one other gene, 9 cases had additional mutations in two other genes and 3 cases had additional mutations in three other genes.

**Table 2:** Genetic alterations in AML patients with/without *IDH1* mutations.

Parameters	AML	R132 IDH1-mutated	Non-R132 IDH1-mutated	AML (IDH1 wild type)	P-value (R132 v wt)	P-value (non-R132 v wt)
AML1,ETO	44	0	2(4.55%)	42(95.45%)	0.042	0.305
PML-RARA	25	0	3(11.54%)	23(88.46%)	0.194	1
CBFB-MYH11	12	0	0	12(100%)	0.491	0.491
MLL fusions	56	0	4(7.14%)	52(92.86%)	0.009	0.433
FLT3-ITD	14	1(7.14%)	6(42.86%)	7(50%)	1	0
NRAS mutation	32	0	7(21.88%)	25(78.12%)	0.165	0.042
NPM1 mutation	15	1(6.67%)	3(20%)	11(73.33%)	1	0.32
CEBPA	5	0	3(60%)	2(40%)	1	0.003
RUNX1	11	0	1(9.09%)	10(90.91%)	0.591	1
KIT	102	0	8(7.84%)	94(92.16%)	0	0.382
WT1	30	3(10%)	19(63.33%)	8(26.67%)	0.154	0
MN1	12	2(16.67%)	6(50%)	4(33.33%)	0.116	0
FLT3	78	0	12(15.38%)	66(84.62%)	0.003	0.074
EVI1	37	0	5(15.31%)	32(86.49%)	0.094	0.648
PRAME	14	0	4(28.57%)	10(71.43%)	0.591	0.057

### 6. *IDH1* mutations and prognosis

**Figure 3:**
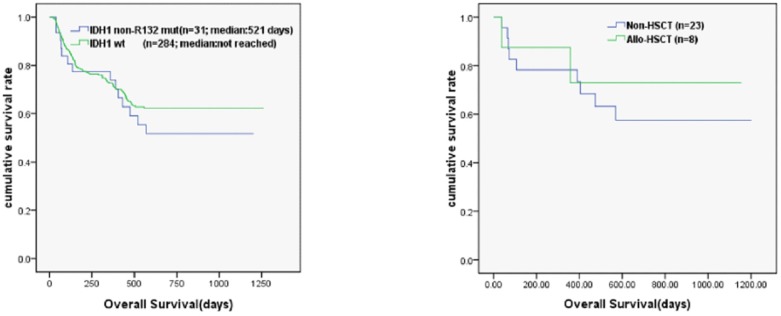
Survival curve of AML patients. Kaplan-Meier curve shows OS (A) in patients with IDH1non-R132 mutations and IDH1 wild type. The OS (B) in patients with non-R132 IDH1 mutations who were transplanted or non-transplanted.

Thirty-four patients were lost to attrition during follow-up and 315/349 cases had final follow-up. Figure 3a shows that the OS in patients with *IDH1* non-R132 mutations was not statistically significant from that of patients with wild type *IDH1* (median 521 days vs median not reached; n.s.). When the patients with *IDH1* non-R132 mutations were grouped on the basis of having received a transplant, the OS (Fig. 3b) in the transplanted group was higher than in the untransplanted group, but they were not statistically different (*n.s.*).

## Discussion

The frequency of *IDH1* mutations in AML patients in Western countries was 2–14%. Our analysis of *IDH1* mutations in 349 newly diagnosed Chinese AML patients, revealed a mutation frequency of 10.03% (35/349). In contrast, four previous reports of *IDH1* mutations in Chinese populations determined an *IDH1* mutational rates of 5.5% (27/493)[18], 5.9% (4/68)[19], 6.3% (23/365)[20] and 2% (4/198)[21] (Table 3). The discrepancies between the mutational rates in the Chinese studies and our current study may be related to differences in selection criteria, detection approach, deviation processing and the north-south racial differences. In addition, these previous studies from China focused on known *IDH1* mutations, while we identified five mutations that new mutations in AML.

**Table 3:** Summary of clinical characteristics in IDH1 mutation patient.

Studies	AML, no	IDH1 R132 mut, no	IDH1 non-132 mut, no	Association	Survival
WenChien Chou	493	27	0	FAB M1, normal CG, NPM1 mut	No effect
Zou Y	68	3	1	No analysis	No survival analysis
Zhang Y	365	23	0	No correlation	shorter DFS
Lin J	198	4	0	older age, normal CG	No effect
This study	349	4	31	IDH1 non-132 mut: FLT3-ITD, CEBPA, and NRAS, as well as the overexpression of MN1 and WT1	No effect

In the present study we identified eight *IDH1* mutations, five of which were not previously described in AML. Among these 5 mutations, the R100Q mutation was localized at Mg^2+^ and the substrate recognition site of isocitrate dehydrogenease complex. Along with the amino acids at R132 and R109, it forms a salt bridge that is required for substrate binding. A previous study in glioma suggested higher 2-HG levels as a result of a mutation at R100[22]. To date, there were only two reports or I99M in Asian populations [19], [23]. Although I99M is not a substrate binding site, it is in close proximity to R100 and the substrate biding region, which supports the notion that it may play a role in AML pathogenesis. Further studies are needed to elucidate the role of these mutations in AML. On the other hand, consistent with previous studies of Asian populations, the present study has found that although the frequency of *IDH1* mutations in China was high, the frequency of R132 mutation was low[16], [18]. This was in stark contrast to reports from Western countries, suggesting potential ethnic diversity in *IDH1* mutations. Taken together, these data have strong implications for targeted therapeutic drugs. For example, in Chinese populations, the incidence of *IDH1* R100 was remarkably higher than that of R132, suggesting that *IDH1* R100 should be a primary experimental target for Chinese or Asian patients.

*IDH1* mutations typically occur in normal karyotype and standard risk karyotype patients. However, our results have showed that the frequency of *IDH1* mutations in our patient population was not different between abnormal and normal karyotypes. In fact, we found a correlation between *IDH1* mutations and other genetic abnormalities, such as *FLT3-ITD* mutation, *NRAS* mutation, *CEBPA* mutation, *WT1* overexpression and *MN1* overexpression. Moreover, *IDH1* mutations were not correlated with *NPM1* mutation. The results reported in Western countries showed a correlation between *IDH1* mutations and *NPM1* mutation. Therefore, further studies of larger Asian populations are needed to determine the frequency and clinicosignificance of *IDH1* mutations combined with *NPM1* mutations in Asian AML patients.

The oncogenic mechanisms of *IDH1* mutations are not fully understood. It is possible that increased 2-HG levels could inhibit α-KG-dependent enzymes, such as the histone demethylation enzymes with JmjC domain and the *TET* family DNA hydroxylases. These enzymes mediate histone and genomic methylation, which impact epigenetics and gene expression profile, thus leading to abnormal cell differentiation and oncogenesis. This putative role for *IDH1* mutations and 2-HG-dependent upregulation of histone demethylation enzymes and *TET* family DNA hydroxylases is supported by the correlation between *IDH1* mutations and the overexpression of *WT1* and *MN1*.

With respect to clinical features, there were no differences in age, leucocyte count, hemoglobin, platelet count and blast myeloid cells between patients with *IDH1* mutations and wild type group. We did observe a higher rate of *IDH1* mutations in males relative to females. Consistent with previous report, *IDH1* mutations occurred most frequently in AML-M_1_.

Owing to low number of *IDH1* mutated cases of *IDH1* R132 mutations in our study, the influence of which on OS could not be discussed here. There was no statistically significant difference between three-year OS in patients with *IDH1non-R132* mutations and patients without *IDH1* mutations. These findings of OS were not statistically significantly different between the groups. When the patients with *IDH1 non-R132* mutations were subgrouped based on whether they received a transplant, the OS in the transplanted group were higher than that in untransplanted group; however, there was no statistically significant difference between the two groups. While is appeared that the prognosis in *IDH1 non-R132* mutation group was better than that in the wild type group, they were not statistically significantly different. Therefore, our findings that *IDH1 non-R132* mutations do not impact the prognosis of Chinese patients with AML, are consistent with previous reports of Asian populations. When the *IDH1 non-R132* mutation group was further subgrouped based on the basis of transplanted or nontransplanted, the prognosis was better in the transplanted group than that in the untransplanted group. The failure to achieve statistical significance was likely due to the small sample size.

## Conclusion

We investigated the *IDH1* mutational status in 349 newly diagnosed Chinese AML patients. *IDH1* mutations were identified in 35 (10.03%) cases. Five *IDH1* mutations were discovered in AML patients for the first time in AML, including 4 *IDH1* R132 mutation and 31 *IDH1* non-R132 mutations. *IDH1non-R132* mutations occurred most frequently within M_1_. There were no statistically significant differences in incidence of *IDH1 non-R132* mutations between normal and abnormal karyotypes. The *IDH1 non-R132* mutations may accompany with *FLT3-ITD*, *NRAS* and *CEBPA* mutations as well as *WT1 and MN1* overexpression. The OS in patients with *IDH1 non-R132* mutations was higher than that of patients with wild type *IDH1*; however, this was not statistically significantly different.

## Supporting Information

Figure S1 **The Sanger traces for all novel IDH1 mutations.** DNA sequencing chromatograms of *IDH1* mutations in matched tumor and remission samples.
(TIF)

Table S1 **PCR primers for mutated genes.**
(DOC)

Table S2 **PCR primers and probes for six genes.**
(DOCX)
