# Supplementary figures and images for: The Frequency and Clinical Significance of IDH1 Mutations in Chinese Acute Myeloid Leukemia Patients
Source: PLoS One. 2013 Dec 20;8(12):e83334. doi: 10.1371/journal.pone.0083334 (PMC3869765; doi:10.1371/journal.pone.0083334)

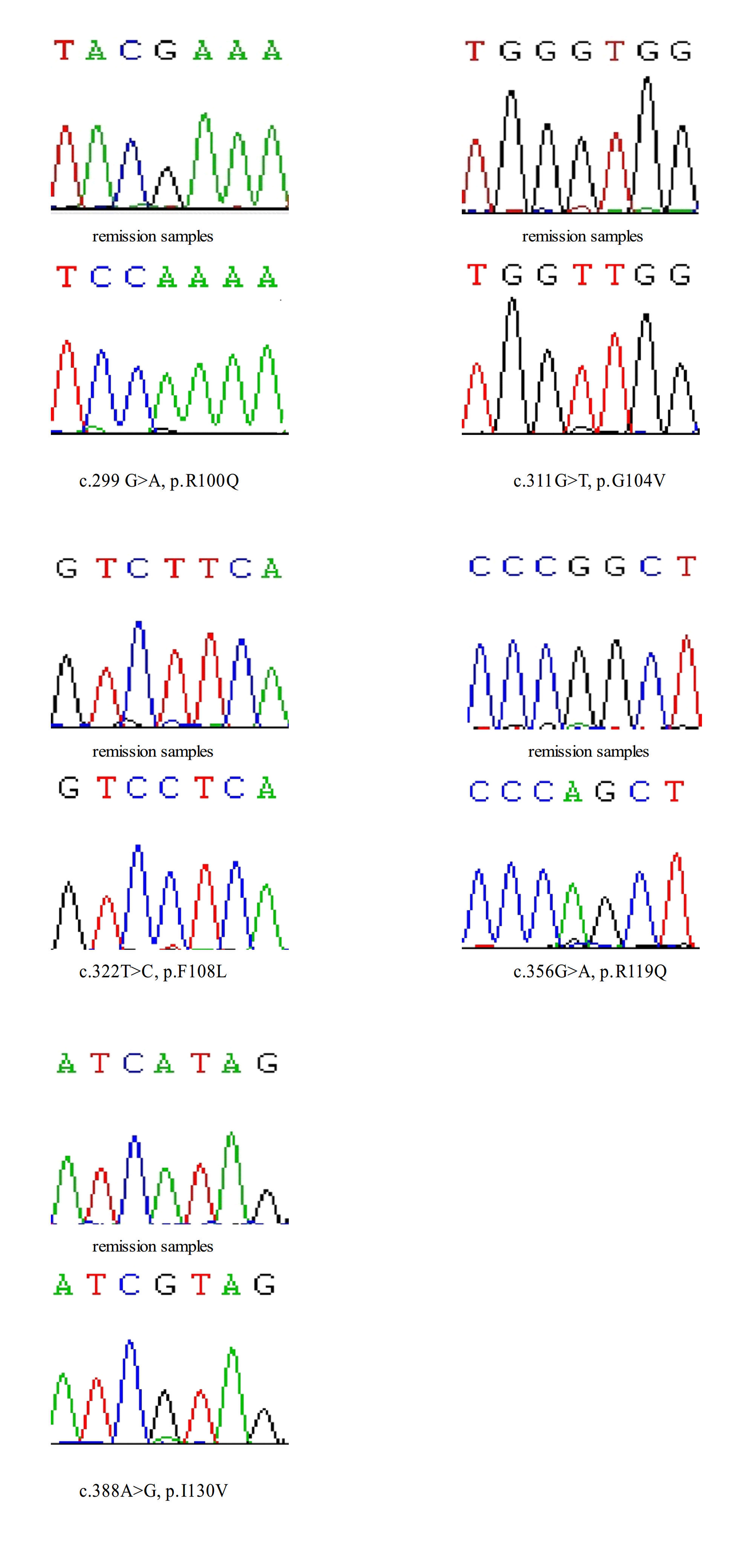

Supplement: Figure S1 — The Sanger traces for all novel IDH1 mutations. DNA sequencing chromatograms of IDH1 mutations in matched tumor and remission samples. (TIF) [file pone.0083334.s001.tif]
