# Supplementary material for: The Frequency and Clinical Significance of IDH1 Mutations in Chinese Acute Myeloid Leukemia Patients
Source: PLoS One. 2013 Dec 20;8(12):e83334. doi: 10.1371/journal.pone.0083334 (PMC3869765; doi:10.1371/journal.pone.0083334)
Supplement: Table S1 — PCR primers for mutated genes. (DOC) [file pone.0083334.s002.doc]

**Supplementary table 1**

**PCR primers for mutated genes**

| No. | Genes | Primers (5’-3’) | |
| --- | --- | --- | --- |
|  |  | Forward | Reverse |
| 1 | *NRAS* | ATGACTGAGTACAAACTGGTGGTGG | CAACCCTGAGTCCCATCATCA |
| 2 | *MLL* | AAGAGTGCCTTGACGATACAGCTA | TTCACACAGCCAGGAGTCTTTTC |
| 3 | *NPM1* | AAAAGGTGGTTCTCTTCCCAAAG | AAAGGACAGCCAGATATCAACTGTT |
| 4 | *CEBPA* | GCAGGCGGTCATTGTCACT | GCAGGCGGTCATTGTCACT |
| 5 | *FLT3* | GACCTGGAAGAAGTGTTCAGACAA | AGTTCTGACATGAGTGCCTCTCTTT |
| 6 | *IDH1* | TCGTGATGCCACCAACGA | ACCTTTTGGGTTCCGTCACTT |
| 7 | *RUNX1* | AGAAGCTGGGTTTGACCAAAGA | GTGGCTTTTCGGTGTGAACA |
