# Supplementary material for: The Frequency and Clinical Significance of IDH1 Mutations in Chinese Acute Myeloid Leukemia Patients
Source: PLoS One. 2013 Dec 20;8(12):e83334. doi: 10.1371/journal.pone.0083334 (PMC3869765; doi:10.1371/journal.pone.0083334)
Supplement: Table S2 — PCR primers and probes for six genes. (DOCX) [file pone.0083334.s003.docx]

**Supplementary table 2**

**PCR primers and probes for six genes**

| No. | Genes |  | |
| --- | --- | --- | --- |
|  |  | Primers (5’-3’) | Probes |
| 1 | *WT1* | F GCTATTCGCAATCAGGGTTACAG  R GGGATCCTCATGCTTGAATGA | ACGGTCACCTTCGACGG |
| 2 | *KIT* | F AGGGCCACCGTTTGGAAAG  R TTACATTCAACCGTGCCATTG | TAGTGGTTCAGAGTTCTATAG |
| 3 | *FLT3* | F GGCCGTGTTTCGGAATGTC  R CGGAGAGAGTAGCCCCAAATC | AGGCGACCTTTCAGCAG |
| 4 | *EVI1* | F ACTTAGCCGTTCACTCCGCTAT  R CCATGGAGAAAAGGGCTTCTG | ACTATGAGAAAGGAATTATG |
| 5 | *PRAME* | F CATCTTCACCTGGAGACCTTCAA  R TTCTTCCGTAAATCCAGCACTTG | TGTGCTTGATGGACTTG |
| 6 | *ABL1* | F CTCCATTATCCAGCCCCAAA  R CCCAGCTTGTGCTTCATGGT | CGCAACAAGCCCACTG |
